# The German Cancer Consortium (DKTK) multi-center prospective phase 1/2 ^68^Ga-PSMA-11 PET-imaging trial in newly-diagnosed high-risk prostate cancer: Safety and diagnostic accuracy compared to histopathology and their impact on patient management

**DOI:** 10.1007/s00259-025-07540-4

**Published:** 2025-11-15

**Authors:** Frederik L. Giesel, Stefan A. Koerber, Boris Hadaschik, Selina Kiefer, Sarah Schwarzenboeck, Kambiz Rahbar, Thorsten Derlin, Christoph A. Grott, Matthias Heck, Cordula Jilg, Philipp T. Meyer, Juri Ruf, Karl Schmidt, Joerg Kotzerke, Christian la Fougère, Gerald Reischl, Irene Virgolini, Ken Herrmann, Irene A. Burger, Theo Lorenzini, Martin Werner, Albrecht Stenzinger, Kristina Schwamborn, Jan Philipp Radtke, Markus Hohenfellner, Juergen Debus, Wolfgang A. Weber, Uwe Haberkorn, Klaus Kopka, Matthias Eiber, 68Ga-PSMA-11 DKTK trial contributors, Ursula Sahm, Steffen Rausch, Clemens Decristofero, Christian Uprimny, Gianpaolo di Santo, Christine Rangger, Gerd Wunderlich, Johannes Huber, Ulrich Sommer, Anass Johayem, Philipp A. Kaufmann, Daniel Eberli, Eric Tonndorf-Martini, Mathias Muehlhauser, Clemens Kratochwil, Kai Schubert, Oliver Kiss, Ute Hennrich, Peter Choyke, Claus Zippel, Otmar Wiestler, Michael Baumann, Annette Reil-Held, Volker Meyer, Sandra Meese, Tobias Maurer, Marianne Remke, Petra Watzlowik, Franz Gildehaus, Olaf Prante, Alexander Höpping, Gesine Kabuss, Jürgen Gschwend, Gabriele Kienzle

**Affiliations:** 1 Department of Nuclear Medicine, Heidelberg University Hospital, Heidelberg, Germany; 2 Department of Nuclear Medicine, Medical Faculty and University Hospital Duesseldorf, Heinrich-Heine-University Duesseldorf, Duesseldorf, Germany; 3 Department of Radiation Oncology, Barmherzige Brüder Hospital Regensburg, Regensburg, Germany; 4 Department of Radiation Oncology, Heidelberg University Hospital, Heidelberg, Germany; 5 Department of Urology, University Hospital Essen, Essen, Germany; 6 Institute for Surgical Pathology, Medical Center—University of Freiburg, Faculty of Medicine, University of Freiburg, Freiburg im Breisgau, Germany; 7 Department of Nuclear Medicine, University Medical Centre, Rostock, Germany; 8 Department of Nuclear Medicine, Münster University Hospital, Münster, Germany; 9 Department of Nuclear Medicine, Hannover Medical School, Hanover, Germany; 10 Department of Urology, TUM University Hospital, School of Medicine and Health, Technical University Munich, Munich, Germany; 11 Department of Urology, Faculty of Medicine, University of Freiburg—Medical Centre, Freiburg, Germany; 12 Department of Nuclear Medicine, Medical Center–University of Freiburg, Faculty of Medicine, University of Freiburg, Freiburg, Germany; 13 ABX-CRO, Dresden, Germany; 14 Department of Nuclear Medicine, Faculty of Medicine and University Hospital Carl Gustav Carus, Technische Universität Dresden, Dresden, Germany; 15 Department of Nuclear Medicine and Clinical Molecular Imaging, University Hospital Tuebingen, Tuebingen, Germany; 16 Department of Preclinical Imaging and Radiopharmacy, University Hospital Tuebingen, Tuebingen, Germany; 17 Department of Nuclear Medicine, University Hospital Innsbruck, Innsbruck, Austria; 18 Department of Nuclear Medicine, West German Cancer Center, University Hospital Essen, Essen, Germany; 19 Cancer Consortium partner site Essen/Düsseldorf, DKFZ and University Hospital Essen, Essen, Germany; 20 Department of Nuclear Medicine, Kantonsspital Baden, Affiliated Hospital for Research and Teaching of the Faculty of Medicine of the University of Zurich, Baden, Switzerland; 21 Department of Nuclear Medicine, University Hospital Zurich, University of Zurich, Zurich, Switzerland; 22 Department of Nuclear Medicine, TUM University Hospital, School of Medicine and Health, Technical University Munich, Munich, Germany; 23 German Cancer Consortium (DKTK), Munich, Germany; 24 Institute of Pathology, Division of Molecular Pathology, University Hospital Heidelberg, Heidelberg, Germany; 25 Institute of Pathology, Technical University of Munich, School of Medicine, Munich, Germany; 26 Department of Urology, Medical Faculty, University Duesseldorf, Düsseldorf, Germany; 27 Department of Urology, University Hospital Essen, Essen, Germany; 28 Department of Urology, University Hospital Heidelberg and National Center for Tumor Diseases (NCT) Heidelberg, Heidelberg, Germany; 29 Center for Translational Cancer Research (TranslaTUM), Technical University of Munich, Munich, Germany; 30 Bavarian Cancer Research Center (BZKF), Munich, Germany; 31 Division of Radiopharmaceutical Chemistry, German Cancer Research Center, Heidelberg, Germany; 32 Helmholtz-Zentrum Dresden-Rossendorf e.V., Institute of Radiopharmaceutical Cancer Research, Dresden, Germany; 33 German Cancer Consortium (DKTK), Heidelberg, Germany; 34 Institute for Radiation Sciences, Osaka University, Osaka, Japan

**Keywords:** ^68^Ga-PSMA-11, PET, High risk prostate cancer, Safety, Diagnostic accuracy, Radiation therapy planning

## Abstract

**Purpose:**

Clinically accurate detection of prostate cancer (PCa) metastases is crucial for management of high-risk PCa patients scheduled for radical prostatectomy. We determine the safety and diagnostic accuracy of pre-operative ^68^Ga-PSMA-11 PET/CT imaging in newly diagnosed high-risk PCa and assess its impact on patient management.

**Methods:**

Investigator-initiated prospective multi-center multinational single-arm open-label phase 1/2 imaging trial (EuRadCT 2016–001815-19). Patients with high-risk PCa scheduled for prostatectomy were enrolled at 9 institutions in Germany, Austria, and Switzerland to undergo ^68^Ga-PSMA-11 PET/CT for primary staging.

The primary objectives were the evaluation of ^68^Ga-PSMA-11 PET/CT imaging to detect the primary tumor and lymph node disease and safety assessment. Secondary objectives included detection of distant metastases, correlation of ^68^Ga-PSMA-11 uptake with Gleason Score, and determining the impact on clinical management. Impact of pre-operative ^68^Ga-PSMA-11 PET/CT imaging on target volume definition for radiation therapy was assessed.

**Results:**

173 patients underwent ^68^Ga-PSMA-11 PET/CT for primary staging. Histopathologic correlation was available in 139 patients (imaging dataset), with lymph node metastases in 55 patients (39.6%). 20 treatment-emergent AEs unrelated to the test item were reported in 14 of 173 (8.1%) patients and no SAE occurred. On a per-patient basis, sensitivity of ^68^Ga-PSMA-11 PET for local disease was 0.971 (95% CI, 0.928–0.992). Sensitivity, specificity, PPV, NPV and accuracy to detect local lymph node disease on a per-patient basis were 0.400 (95% CI 0.271–0.529), 0.988 (95%CI 0.965–1.000), 0.957 (95% CI 0.873–1.000), 0.716 (95% CI 0.633–0.798) and 0.755 (95% CI 0.684–0.827), respectively. Considering the intrinsic PET resolution of 3–5 mm, the exclusion of lesions smaller than 3 or 5 mm on histopathology from the analysis led to increased sensitivity of 56.4% and 69.0%, respectively. Median SUVpeak of local disease was 6.4 (range 1.7–13.6), 8.4 (range 2.3–39.4), 10.7 (range 5.6–23.0), and 13.4 (range 3.8–56.9) for Gleason Score 7a, 7b, 8 and 9, respectively. Based on the results of ^68^Ga-PSMA-11 PET/CT, surgical intervention was canceled in 23 patients (13.2%). ^68^Ga-PSMA-11 PET/CT resulted in a change of target volume delineation for radiation therapy planning in 29 patients (20.9%).

**Conclusion:**

In high-risk primary PCa, ^68^Ga-PSMA-11 is safe and effective in local staging, resulting in changes in both surgical and radiation management. Moreover, ^68^Ga-PSMA-11 uptake is positively correlated with tumor grade and its efficacy is dependent on the size of nodal lesions. ^68^Ga-PSMA-11 PET/CT will be highly impactful in the management of newly diagnosed high risk prostate cancer patients.

**Funding:**

The study was funded by the German Cancer Consortium (DKTK).

**Supplementary Information:**

The online version contains supplementary material available at 10.1007/s00259-025-07540-4.

## Introduction

Prostate cancer (PCa) is the second most common malignancy in men worldwide [1]. Staging of newly diagnosed PCa aims to detect both regional nodal involvement (N1) and distant metastatic disease (M1), the presence of which dramatically alters management and prognosis. Until recently, bone scintigraphy and abdominal/pelvic computed tomography (CT) or magnetic resonance imaging (MRI) were the mainstay imaging methods for initial staging of newly diagnosed high-risk prostate cancer [2]. However, cross-sectional imaging by CT or MRI for N-staging proved to have limited diagnostic performance: In a meta-analysis conducted by Hövels et al., cross-sectional imaging modalities yielded low sensitivities for lymph node metastases (pooled sensitivity of 0.42 for CT and 0.39 for MRI) [3]. Notably, in a subsequent prospective trial, Briganti et al. reported an even lower overall sensitivity of 0.13 for CT scans [4]. Conventional cross-sectional imaging modalities such as CT have often been paired with bone scans to improve detection of bone metastases.

The advent of prostate-specific membrane antigen (PSMA)-ligand PET/CT imaging has substantially improved prostate cancer staging [5–7]. Both initial retrospective results and prospective trials have demonstrated the ability of ^68^Ga-PSMA-11 to detect the sites of biochemical recurrence and improve the staging of regional and distant disease in intermediate and high risk primary PCa [8–10]. ^68^Ga-PSMA-11 (gallium Ga-68 gozetotide) has gained US Food and Drug Administration (FDA) approval and its success has led to the incorporation of PSMA-ligand imaging in a variety of national and international practice guidelines [2, 11–14]. These ligands, in addition to the aforementioned ^68^Ga-PSMA-11, include ^68^ Ga-PSMA-I&T, ^18^F-DCPyL, ^18^F-rhPSMA-7.3 and ^18^F-PSMA-1007 [13, 15, 16].

^68^ Ga-PMSA-11 PET/CT outperforms other targeted molecular imaging agents, such as ^18^F choline or fluciclovine, providing improved diagnostic accuracy and resulting in profound changes in patient management [8–10, 17, 18]. Findings from the proPSMA trial demonstrate a clear superiority of ^68^Ga-PSMA-11 compared to cross-sectional imaging and bone scan for the staging of high-risk primary PCa [19].

The German Cancer Consortium (DKTK) started an initiative to evaluate this new imaging agent in a prospective multi-national, multi-center phase 1/2 to evaluate safety and diagnostic efficacy in primary high risk prostate cancer. Within this prospective multicenter study, we aimed to assess the diagnostic accuracy of PSMA-PET/CT including patients with newly diagnosed prostate cancer at high risk for metastases who were scheduled for radical prostatectomy (RP) with extended pelvic lymph node dissection (EPLND). Primary objectives were i. the assessment of ^68^Ga-PSMA-11 PET/CT imaging to detect prostate cancer tissue within the prostate gland and pelvic lymph nodes and ii. to assess the clinical safety of ^68^Ga-PSMA-11.

Secondary objectives included i. the ability of ^68^Ga-PSMA-11 PET imaging to detect bone metastases in comparison to ^99m^Tc bone scintigraphy, ii. to correlate ^68^Ga-PSMA-11 uptake in primary tumours with Gleason Score in post-surgical specimens, iii. to determine the percentage of patients in which pre-operative ^68^Ga-PSMA-11 PET/CT imaging would result in a change in clinical management and iv. to evaluate the impact of pre-operative ^68^Ga-PSMA-11 PET/CT imaging on target volume definition for radiation therapy.

## Material and methods

### Study design and participants

A prospective open-label, single-arm, rater-blinded, multinational multi-center, diagnostic phase 1/2 study to assess safety and diagnostic performance of ^68^Ga-PSMA-11 PET/CT imaging to detect sites of prostate cancer in patients with newly diagnosed high-risk PCa (NCT03362359) was performed at 9 academic sites in Germany, Austria and Switzerland.

The study protocol was created in accordance with the Declaration of Helsinki and Good Clinical Practice, and all patients gave written informed consent before study entry. The trial was approved by the local institutional review boards, the Federal Office for Radiation Protection *(Bundesamt fuer Strahlenschutz*) and the Federal Institute for Drugs and Medical Devices (*Bundesinstitut für Arzneimittel und Medizinprodukte*, BfArM). The study drug, ^68^Ga-PSMA-11, was produced under locally established manufacturing allowances (*Herstellungserlaubnis*) harmonized between all centers and approved by the respective local governments. The trial was funded by the German Cancer Consortium (DKTK) and the protocol is provided in the appendix 1. Study and data management was conducted by ABX-CRO, Dresden, Germany.

Eligibility criteria included patients with histologically confirmed adenocarcinoma of the prostate, high risk for metastasis (stage cT3, Gleason Score > 7, or PSA > 20 ng/ml), who were scheduled for surgery (radical prostatectomy and extended lymph node dissection). Patients were excluded if they demonstrated hypersensitivity to ^68^Ga-PSMA-11 or its components, had known lymph node metastases outside the surgical field, > 5 bone metastases (based on ^99m^Tc bone scintigraphy), and any previous prostate cancer therapy. Prior to study inclusion a ^99m^Tc-bone scan and cross-sectional (CE-CT or CE-MRI) imaging of abdomen and pelvis were obtained consistent with EAU guidelines [12].

### Procedures

Each patient received a single intravenous injection of 150 (± 50) MBq ^68^Ga-PSMA-11 on day 1 followed by PET/CT imaging at 1 h ± 10 min p.i. A low-dose CT was performed for attenuation correction followed by a diagnostic contrast-enhanced CT. PET acquisition time for each bed position was 3–4 min. Details on image acquisition and reconstruction are provided in the subject imaging manual in appendix 2 and supplementary materials*.* Please note that both conventional and digital scanners with and without time-of-flight have been used at the different trial sites. Scanners were EARL certified.

### Image interpretation and radiotherapeutic planning

Three independent readers performed a central reading. Each patient scan was evaluated by two independent readers (Reader 1: SS and Reader 2: KR). If the reading results were concordant (i.e. in agreement), this was considered the final reading result. In case of disagreement between the two independent readers, an adjudication reading by a third reader (adjudicator: TD) was performed and became the final reading result (majority vote). All raters were blinded to the results of any histological investigations and preceding imaging (e.g., bone scan, MRI). All readers were experienced on clinical ^68^Ga-PSMA-11 reading and were required to complete training on 12 cases (not part of the present study population). Each reader had to re-assess 10% of the cases for quality assurance purposes.

The following qualitative analyses were performed based on the recently introduced molecular imaging TNM system (miTNM, version 1.0) as a standardized reporting framework for PSMA PET [20]. Tumour involvement of the prostate gland was assessed based on quadrants (right/left, upper/lower part) on the PET/CT image data and was reported on a 3-point scale (positive, equivocal, negative). All pelvic lymph nodes, in eight pre-defined sub-regions, (appendix 2) visible on the PET/CT and diagnostic CT scan were evaluated using a 3-point scale (positive, equivocal, negative). The short axis diameter of each representative lymph node was measured. Quantitative measurement of ^68^Ga-PSMA-11 uptake was performed in the hottest foci of the primary tumor (dominant lesion) using the maximum SUVpeak. Qualitative and quantitative analyses were performed using Mind Lesion (Mind Medical GmbH, Heidelberg).

Fictitious radiotherapy planning delineating target volumes independently based on conventional imaging and ^68^Ga-PSMA-11 PET was performed by experienced radiation oncologists blinded to the histopathological findings (CAG, SAK). Differences in target volume definitions from conventional imaging and PET were determined. SUV-parameters in PSMA-PET imaging for lesion based radiotherapeutic planning and management SUV-parameters were used as recently published [21].

### Safety

Physical examination, vital signs, 12-lead ECG, pulse oximetry and laboratory tests (haematology, serum chemistry, urine analysis) were performed before dosing and 1.5 h ± 30 min, 24 h ± 4 h and 7 d after dosing. All adverse events and concomitant medications were recorded until 7 days after dosing. For details, see study flow chart (appendix 1).

### Surgery, follow-up, and histopathological correlation

Surgery (radical prostatectomy and extended lymph adenectomy) and analysis of tissue specimens was performed as part of the standard care between day 7 (after EOS visit) and day 60. Only information about PSMA-positive lymph nodes outside the extended pelvic node dissection and any additional bone lesions detected by the local nuclear medicine investigator were conveyed to the surgeon to aid in their treatment planning. EPLND was conducted according to the EAU guideline 2015 with 8 sub-regions (supplementary materials). Tissue from each subregion was separately collected. Tissue specimens obtained from RP with EPLND were analyzed blinded to the results of PET imaging, first by the local pathologists and then transferred for a further second central pathology reading.

### Outcomes and variables

Primary endpoints of the study were sensitivity, specificity, positive predictive value (PPV), negative predictive value (NPV), and accuracy of ^68^Ga-PSMA-11 PET to identify tumor tissue in the prostate gland and pelvic lymph nodes using histopathology as the standard of truth. Analyses were performed on a per-patient and per-quadrant basis for lesions in the prostate gland and on a per-patient, per-gross region (pelvic right vs. left) and per-subregion basis for lymph nodes. Safety and tolerability of ^68^Ga-PSMA-11 was assessed in terms of frequency of occurrence and severity of abnormal findings during safety evaluations. Secondary endpoints included detection of bone metastases, quantitative standard uptake values (SUV) stratified by Gleason Score, fraction of patients with change in clinical management and the impact on target volume definition for radiation therapy. In exploratory post-hoc analyses the sensitivity of ^68^Ga-PSMA-11 PET to identify lymph node metastases was stratified by lesion size on histopathology and interobserver variability for ^68^Ga-PSMA-11 PET reporting was investigated.

### Histopathological workup

The lymph node specimens were processed by each of the participating pathology departments according to their corresponding routine protocol. A central pathology core unit was established where slices from all participating centers were collected and evaluated. Histological results from the prostate and all lymph node specimens were assessed by the central pathologist (SK) using light microscopy (Leica DM2500®, Wetzlar, Germany) with 2.5x, 10 × and 40 × objectives. The size and extent of metastatic infiltration in each lymph node metastasis were determined and measured manually in two dimensions with an ocular micrometer. The total number of resected lymph nodes, number of metastatic lymph nodes and size of the lymph nodes and metastases were documented for each resected region.

### Statistical analysis

Sample size calculation for the study was based on prior experience with patients undergoing primary staging prior to RP [22]. A sensitivity of 75% and a specificity of 96% to detect PCA tissue are expected for ^68^Ga-PSMA-11 imaging. For a sample size of *N = *30 LN positive subjects, a 95% confidence interval (CI) of 0.3 (i.e. 60–90%) can be specified for an expected sensitivity of 75%. To recruit *N = *30 LN positive patients a total sample size of *N = *150 subjects will be needed assuming a prevalence of 20% for lymph node metastases in high risk primary PCa [23]. The resulting 120 subjects without lymph node metastases will allow confirmation of a specificity of about 96%, with a 95% CI of < 0.08 (91–99%).

Outcome parameters were assessed descriptively, separately for primary tumours, and lymph nodes. Regions judged as equivocal on imaging were counted as negative for statistical analysis. Data reported included frequency distribution, mean and 95% CI. CI were calculated using the Wilson score interval method. For the dominant intraprostatic lesion the maximum SUVpeak was compared with the highest Gleason Score. The Kruskal–Wallis test was performed to assess differences across Gleason Scores. In an exploratory post-hoc analysis defined cutoffs between 3 and 5 mm based on tumor diameter on histopathology were used to measure sensitivity of ^68^Ga-PSMA-11 PET/CT to detect lymph node metastases on a per-patient basis. Inter-observer agreement was determined using Brennan/Prediger Kappa (supplementary materials).

### Results

**Fig. 1:**
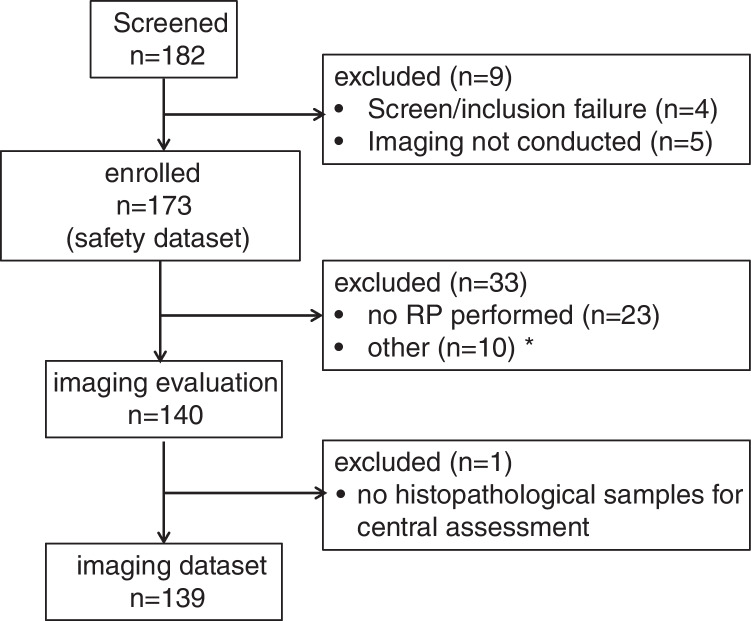
CONSORT diagram. *: start of ADT prior RPE, no DICOM files for bone scan available, other

A total of 182 patients were screened at 9 centers and 173 patients received the study drug (Supplementary Table 1) between 2017 and 2020. All 173 patients were eligible for assessment of safety and tolerability (safety dataset). 140 patients were eligible for image evaluation but one patient lacked histological samples for central assessment. A total number of 139 datasets were eligible for final analysis of the primary imaging endpoints (imaging dataset). The study CONSORT diagram is presented in Fig. 1. The baseline patient characteristics are provided in Table 1*.*

**Table 1:** Characteristics of all patients who received ^68^Ga-PSMA-11 (safety dataset) and subset of patients with correlation of imaging data to histopathology (imaging dataset)

	Safety dataset (*N = *173)	Imaging dataset (*N = *139)
Age, years		
Median Range	66 45–82	66 45–81
Time since prostate biopsy, months		
Median Range	1 0–8	1 0–8
Gleason Score biopsy, n (%)		
6* 7 8 9 10	3 (1.7%) 34 (19.7%) 69 (39.9%) 63 (36.4%) 4 (2.3%)	2 (1.4%) 25 (18.0%) 57 (41.0%) 53 (38.1%) 2 (1.4%)
iPSA, ng/mL		
Median Range	12.2 0.7–204.3	11.3 1.7–204.3
^68^Ga-PSMA-11 PET/CT		
Administered activity, - median (range), MBq	152.6 (89.5–226.0)	153.9 (89.5–226.0)

CT, computed tomography; PET, positron emission tomography; PSA, prostate specific antigen

^*^ all of these patients were classified as high-risk based on PSA > 20 ng/ml

#### Primary endpoints

##### ^68^Ga-PSMA-11 PET imaging to detect prostate cancer tissue within the prostate gland

On a per-patient basis ^68^Ga-PSMA-11 PET/CT was positive by consensus read in 135 patients, resulting in a sensitivity of 0.971 (95% CI, 0.928–0.992). On per-quadrant basis, sensitivity, specificity, PPV, NPV, and accuracy were 0.710 (95% CI 0.670–0.750), 0.600 (95% CI 0.485–0.715), 0.925 (95% CI 0.896–0.952), 0.230 (95% CI 0.196–0.290) and 0.696 (95% CI 0.658–0.734), respectively.

##### ^68^Ga-PSMA-11 PET imaging to detect pelvic lymph node metastases

Lymph node metastases were present at histology in 55 patients (39.6%) from 103 regions. A total of 268 lymph node metastases were identified pathologically, and 28 metastases were outside the standard EPLND field.

**Fig. 2:**
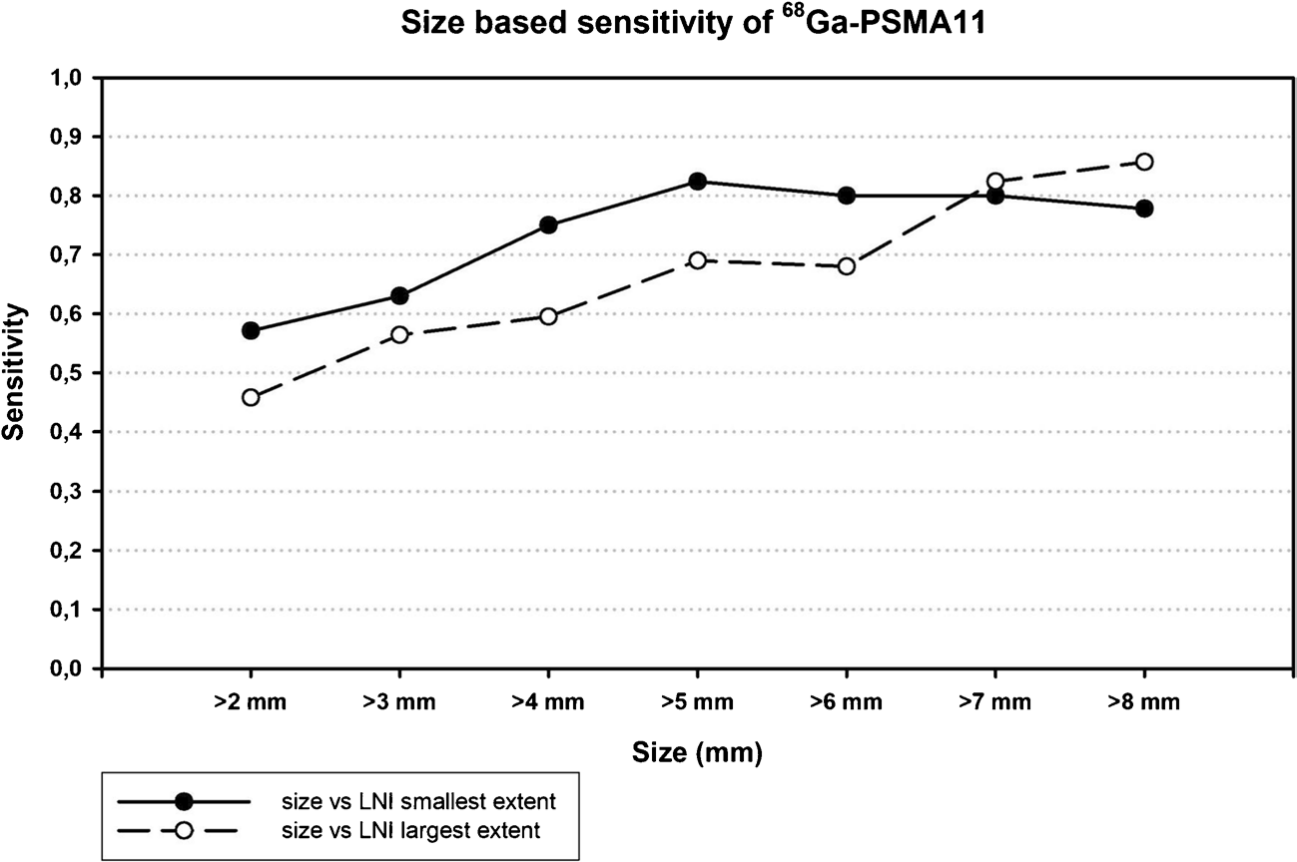
Correlation between lymph node infiltration (LNI) and sensitivity of ^68^Ga-PSMA-11 PET

*On a per-patient basis* sensitivity, specificity, PPV, NPV, and accuracy were 0.400 (95% CI 0.271–0.529), 0.988 (95%CI 0.965–1.000), 0.957 (95% CI 0.873–1.000), 0.716 (95% CI 0.633–0.798) and 0.755 (95% CI 0.684–0.827), respectively. In the exploratory post-hoc analysis, sensitivity was in the range of 0.564 (95% CI 0.396–0.722) for a 3 mm size threshold to 0.690 (95% CI 0.566–0.962) for a 5 mm size threshold of lymph node infiltration (LNI) (Fig. 2)*.* Details on size-stratified PET-diagnostic performance are presented in Supplementary Table 2.

*On a per region hemiplevic (right vs. left pelvis), basis* sensitivity, specificity, PPV, NPV, and accuracy were 0.345 (95% CI 0.244–0.447), 0.995 (95% CI 0.985–1.000), 0.967 (95% CI 0.902–1.000), 0.778 (95% CI 0.727–0.830) and 0.799 (95% CI 0.751–0.846), respectively. *On a per-region or template basis* sensitivity, specificity, PPV, NPV and accuracy were 0.155 (95% CI 0.085–0.225), 0.982 (95% CI 0.973–0.990), 0.471 (95% Ci 0.303–0.638), 0.917 (95% CI 0.900–0.934) and 0.903 (95% CI 0.886–0.921), respectively.

### Inter-reader agreement

In the exploratory post-hoc analysis inter-reader agreement for prostate gland evaluation was 0.90 at patient level and 0.59 at quadrant level. For lymph nodes, the agreement was 0.90 at patient level and 0.93 at sub-region level.

### Safety evaluation

Overall, 20 treatment-emergent AEs were reported in 14 of 173 (8.1%) patients (Table 2)*.* Treatment-emergent AEs were graded as grade 1 (*n = *19) and grade 2 (*n = *1) and none led to study discontinuation. These events were not considered to be related to the study drug. No severe treatment-emergent AEs were reported. Detailed characteristics of AEs are presented in Supplementary Table 3.

**Table 2:** Summary of adverse events

Investigators’ interpretation	Total (*n = *173) (*n* [%])
Total number of TEAEs	20
Patients with at least one TEAE	14 (8.1%)
TEAEs leading to death	0
Total number of SAEs	0
Treatment-related TEAEs	0
Patients with at least one treatment-related TEAE	0
TEAEs leading to discontinuation	0
Patients with at least one TEAE leading to discontinuation	0
TEAEs by toxicity grade	
1	19 (95.0%)
2	1 (5.0%)
3–5	0

#### Secondary endpoints

##### Detection of bone metastases on ^68^Ga-PSMA-11 PET/CT compared to ^99m^Tc bone scintigraphy

12 patients exhibited positive findings by ^68^Ga-PSMA-11 PET/CT and/or bone scintigraphy in 17 anatomic regions. Only in one patient, both imaging modalities were concordantly positive in the same region. Negative ^68^Ga-PSMA-11 PET/CT with positive bone scintigraphy occurred in 7 patients in 7 different regions. Negative bone scintigraphy in ^68^Ga-PSMA-11 PET/CT positive regions was noted in 4 patients with one region in two patients, two regions in one patient and five regions in one patient. Details on patients reported as having positive bone findings are provided in Supplementary Table 4.

##### Correlation of ^68^Ga-PSMA-11 signal with Gleason Score

**Fig. 3:**
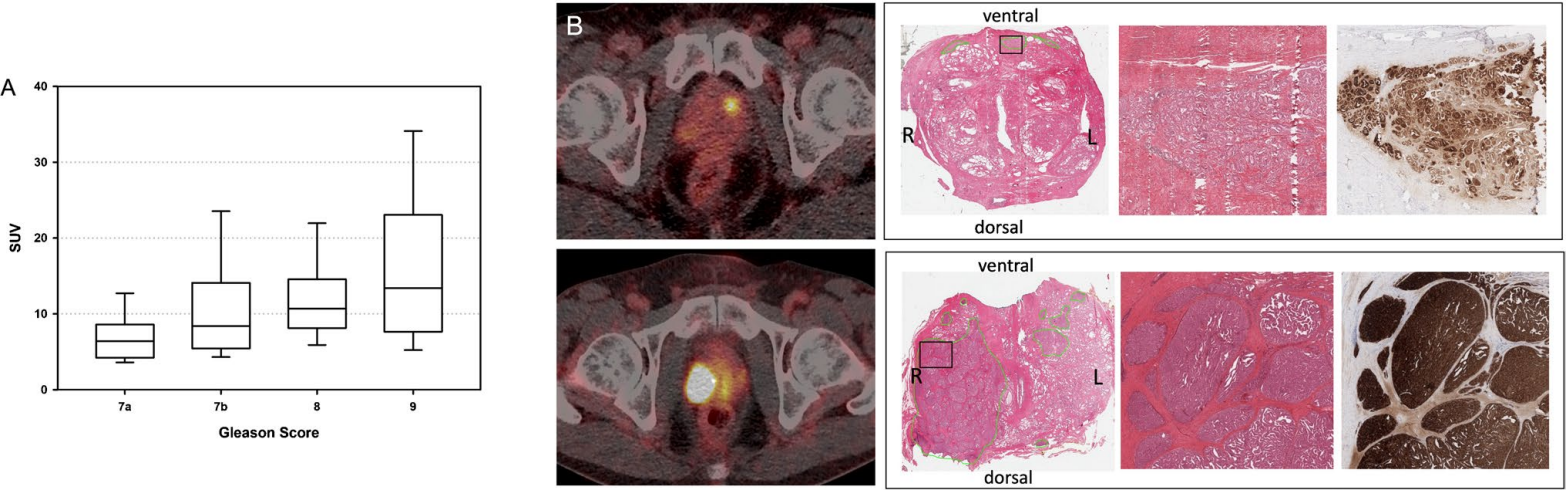
Correlation between ^68^Ga-PSMA-11 uptake and Gleason Score (**A**). PET-imaging, gross-section and detailed histopathology H&E histopathology including PSMA-immunhistochemistry (**B**) of two patients with Gleason Score 7a (upper row) and Gleason Score 9 (lower row)

In 135 of 139 patients, quantitative analyses of ^68^Ga-PSMA-11 uptake in the dominant intraprostatic lesion was performed. Median maximum SUVpeak was 6.4 (range 1.7–13.6), 8.4 (range 2.3–39.4), 10.7 (range 5.6–23.0) and 13.4 (range 3.8–56.9) for Gleason Score 7a, 7b, 8 and 9, respectively. Maximum SUVpeak was significantly different for Gleason Score groups (Kruskal Wallis Test: Chisquare = 21.32, *p* < 0.001, Fig. 3A). Two patient examples are shown in Fig. 3B.

### Fraction of patients with change in clinical management

RP and EPLND were not conducted in a total of 23 patients. Change in clinical management resulted from the presence of unknown metastases first seen on ^68^Ga-PSMA-11 PET/CT precluding the patient from surgery in 11 cases (extensive pelvic lymph node metastases *n = *3; distant lymph node metastases *n = *3; unknown bone metastases *n = *3, pulmonary lesions *n = *2). 5 (21.7%) patients refused surgery, 4 (17.4%) patients received other therapies and 3 patients were lost to follow-up.

### Change of (Potential) radiotherapeutic management

**Fig. 4:**
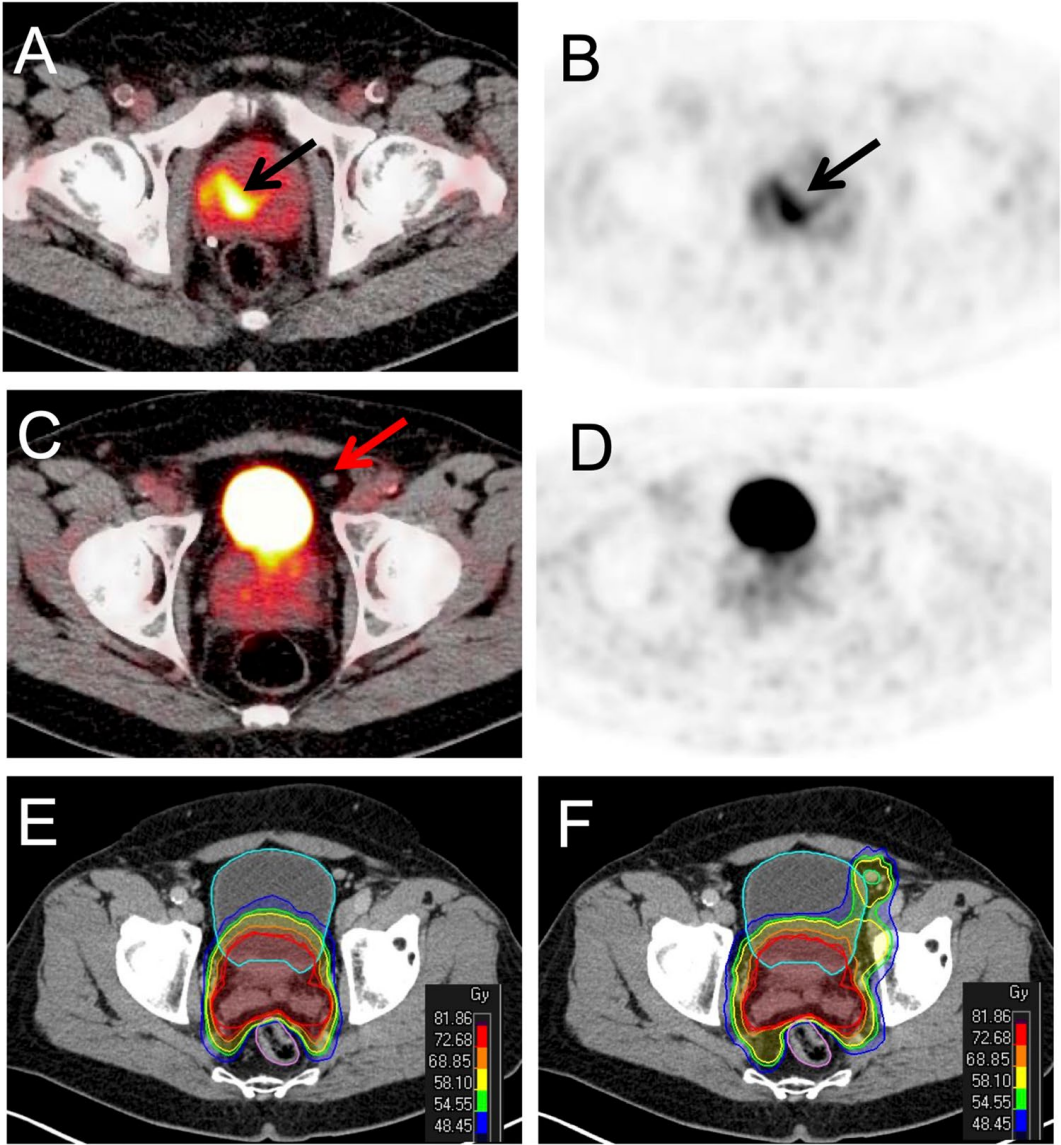
^68^Ga-PSMA-11 PET in a 68 year old patient with a histopathological proven Gleason 8 prostate cancer (iPSA 50 ng/ml). Fused PET/CT and PET-images show intense tracer uptake in the primary tumor (**A** and **B**). A paravesical lymph node (arrow) is suspicious on morphological imaging but negative in ^68^Ga-PSMA-11 PET (**C** and **D**). CT-guided (**E**) vs. ^68^Ga-PSMA-11-PET guided (**F**) radiation therapy planning: extended irradiation due to suspected paravesical node (**E**) in CT; prostate irradiation only (**F**)

**Fig. 5:**
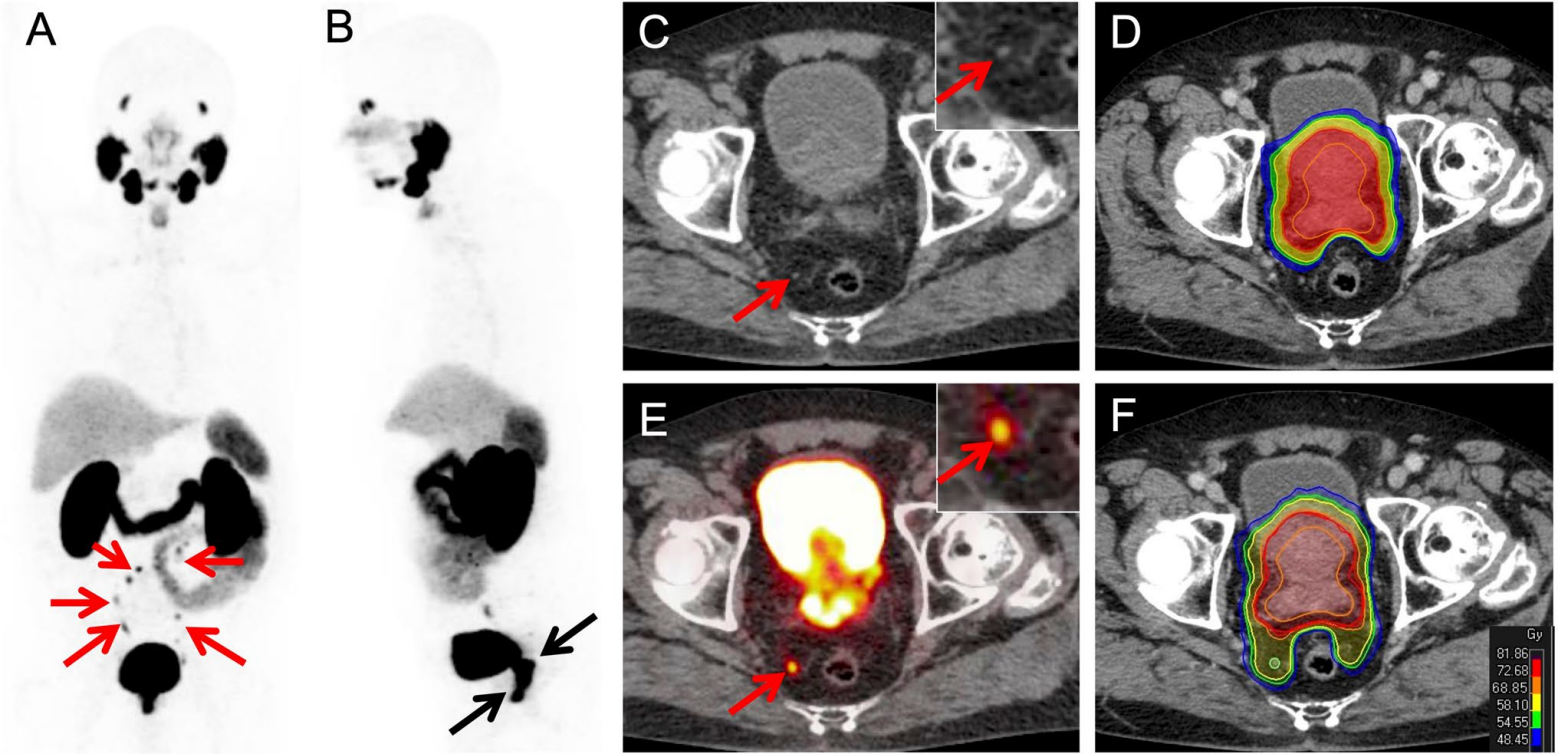
^68^Ga-PSMA-11 PET in a 72 year old patient with a histopathological proven Gleason 7 prostate cancer (iPSA 52 ng/ml). Coronal and sagittal PET MIP-views (**A** and **B**) present intense uptake of the tracer in the primary tumor (black arrow) and multiple pelvic areas with ^68^Ga-PSMA-11 uptake indicating lymph node metastases (red arrows). A small sized pararectal lymph node (4 mm in CT, C) show intense ^68^Ga-PSMA-11 uptake (**E**). CT-guided (**D**) vs. ^68^Ga-PSMA-11 PET guided (**F**) radiation therapy planning: irradiation of the pararectal lymph node was not included (**C**) in CT compared to ^68^Ga-PSMA-11 PET based planning (**F**)

Radiotherapeutic planning was conducted in all patients from the imaging dataset. ^68^Ga-PSMA-11 PET resulted in target volume changes in 29 patients out of 139 patients (20.9%): In 23 patients major changes of the radiation plan occurred. This included upstaging to N1-disease, nodes outside international target volume recommendations (RTOG) and downstaging of morphological suspicious findings. In six patients minor changes were implemented in the radiation plan by altering the number of boosts to nodes based on positive findings in ^68^Ga-PSMA-11 PET/CT (Details see Table 3). Two patient examples are presented in Fig. 4 and 5.

**Table 3:** Change of radiotherapeutic management

Major changes (*n = *23 patients)	N1 instead of N0	*n = *12*
	N0 instead of N1	*n = *7*
	positive node(s) outside RTOG target volume recommendations	*n = *5*
	negative node(s) outside RTOG target volume recommendations	*n = *5*
	M0 instead of M1a	*n = *1*
	M1a instead of M0	*n = *1*
	M1b instead of M0	*n = *2*
Minor changes (*n = *6 patients)	additional SIBs	*n = *5
	different SIBs	*n = *1

*SIB* simultaneous integrated boost (for positive nodes)

^*^for some patients more than one change occurred

### Discussion

In this multicenter prospective phase 1/2 imaging trial in primary high risk PCa,^68^Ga-PSMA-11 proved to be a safe agent that produced highly impactful results. Using independent central reads ^68^Ga-PSMA-11 PET/CT was positive in 97.1% of primary tumors. Sensitivity and specificity on a per-patient basis for the detection of pelvic nodal metastases validated by histopathology were 40.0% and 98.8%, respectively consistent with the findings of other studies of PSMA PET/CT in similar populations. Adjusted to a threshold of 3 and 5 mm, which is the usually physical resolution of a PET-scanner, sensitivity increased to 56.4% and 82.4%, respectively. Change of clinical management, including surgical and radiation planning was observed in a substantial number of patients. To our knowledge, this study is the largest prospective multinational multi-center study using ^68^Ga-PSMA-11 PET focusing on primary high-risk PCa and using extensive centralized histopathological review with analysis on the basis of nodal templates as well as on the basis of LN lesion size.

The predefined specificity of 96% for lymph node disease was exceeded on a per-patient basis. However, the predefined sensitivity of 75% for detection of lymph node metastases was not reached. In retrospect the sensitivity was overestimated as it was based on older data [22]. Emerging data in the literature in primary PCa suggests a sensitivity for lymph node involvement of approximately 40% against the gold standard of careful histological evaluation, which reflects the much higher sensitivity of histopathology for microscopic and even small macroscopic lymph node involvement [14, 24, 25]. Our study is in line with several retrospective and prospective studies: For instance, Hope et al. conducted a prospective phase 3 trial in patients with intermediate and high-risk prostate cancer and reported a sensitivity of 40.0% for lymph node involvement on PET similar to our results [24]. In the surgery cohort of this study, 225 of 277 patients had high-risk disease. A recent meta-analysis by Petersen et al. summarizing the experience of eighteen retrospective and prospective studies reported a weighted sensitivity of 59% (range 23–100%) [26]. Most of the early studies were characterized by small sample sizes and were conducted as single-center retrospective studies lacking blinded independent central readers with matched histopathologic correlation. Such studies will tend to overestimate performance [27].

A major reason for this lower than expected sensitivity is that lymph node metastases are often microscopic and challenge the physical detection limit of PET technology. When patients with lymph node lesions smaller than 3 mm or 5 mm were removed from the dataset, the patient-based sensitivity rose to 56.4 and 69.0%, respectively. Consequently, the “diagnostic window” to detect lymph node metastases in PET imaging begins with lesions approximately 5 mm which compares very favorably to conventional cross-sectional imaging, for which a size of at least 8–10 mm is needed to identify positive lesions [3, 4, 28]. Even then, conventional cross sectional imaging lacks specificity for lymph node metastases as hyperplastic nodes can lead to false positives. The superiority of ^68^Ga-PSMA-11 PET compared to cross-sectional imaging was also confirmed in the proPSMA trial in which a 32% higher area under the curve (AUC) for detection of lymph node metastases was observed with PET vs. conventional imaging, although histology was not used as a gold standard in that study [19].

In the current study, a limitation of this post-hoc analysis of lymph node lesion size threshold is the decreasing number of patients in each size strata. For instance, at the 5 mm threshold only 29 of 55 patients were evaluable, reducing the statistical power. This is, in part, balanced by the higher prevalence of lymph node metastases in our study compared to the literature-based estimate. The lymph node metastasis rate in this study was 39.6%, approximately double the literature estimate of 20%.

The sensitivity of ^68^Ga-PSMA-11 PET/CT for lymph node metastases is also subject to sampling bias. For instance, if the analysis is conducted on a hemi-pelvic or template-based format, sensitivity falls to 34.5% and 15.5%, respectively, as the number of subregions increases the likelihood of sub-detectable lesions within those subregions and further reducing sensitivity. In addition, especially for template-based analysis, errors can result from mismatches between the nodes labeled during surgery and the central reading. This is reflected in the higher number of false positive lesions using the template-based analysis indicated by a drop in PPV from 71.6% in the patient-based analysis to 47.1% in the template-based analysis. From a practical point of view, the patient-based analysis is most useful to the clinician as it determines N-status. However, the template-based analysis heightens awareness of the high likelihood of sub-detectable lymph node metastases even in a high-risk population. The clinical implications in terms of overall survival of sub-detectable lymph node metastases needs to be established in larger prospective studies.

While false negative lymph node findings are a recognized limitation of ^68^Ga-PSMA-11 PET/CT, false positives were relatively unusual, suggesting that a positive scan can be relied upon as an indicator of advanced disease in most cases. ^68^Ga-PSMA-11 uptake was seen in nearly all (97.1%) of primary prostate tumors. These data confirm prior retrospective evidence and indicates ^68^Ga-PSMA-11 PET/CT can be considered a non-invasive imaging biomarker similar in accuracy to immunohistochemistry [22, 29, 30]. High ^68^Ga-PSMA-11 uptake in high-risk prostate cancer is significantly correlated with Gleason Score but shows high inter-patient variability. Thus, it cannot replace Gleason scoring but may indicate the inherent biologic variability within each Gleason score stratum and suggests added potential clinical value for lesion characterization, prognostication and targeted biopsy/treatment [21, 31].

While ^68^Ga-PSMA-11 PET/CT was highly accurate on a per-patient basis, at a more granular level of analysis, mismatches between PET and histology became more clear. Potential reasons include misregistration between imaging and histopathology, small volume disease undetectable by PET, and known physiological background uptake of ^68^Ga-PSMA-11, especially in the central-zone of the prostate gland [32]. Similar to prior retrospective and prospective investigation inter-reader agreement was substantial (> 0.6) for local and nodal reads, confirming the high reproducibility of ^68^Ga-PSMA-11 PET imaging and differentiating it from prostate MRI where inter-reader agreement is substantially lower [10, 24, 33].

Although limited by sample size, the correlation of the ^68^Ga-PSMA-11 PET/CT findings with bone scintigraphy were surprisingly poor. It should be noted that patients with extensive bony metastases (> 5 lesions) were excluded from the study. Thus, in this select group of patients with a small number of bone lesions, most regions with positive signals on bone scintigraphy were negative on ^68^Ga-PSMA-11 PET/CT, while regions positive on PET/CT were often negative on bone scintigraphy. Lacking a definitive standard of reference, it is impossible to compare the accuracy of the two methods. However, retrospective data and especially prospective data from the ProPSMA trial have shown that positive findings on ^68^Ga-PSMA-11 PET/CT are reliable indicator of bone metastases whereas lesions seen only on bone scintigraphy have a high false positive rate [19, 34, 35].

The clinical impact of ^68^Ga-PSMA-11 PET/CT is clearly demonstrated in this study. Planned surgery was not performed in 23 patients (13.2%). In 11 of 23 extra-prostatic disease was depicted on PSMA PET/CT. Similarly, there was a substantial impact on radiotherapeutic management. In 29 of 139 (20.9%) of patients, changes in the radiation plan including alterations in the sites of boosts occurred. Of note, 28 metastases on ^68^Ga-PSMA-11 PET/CT were disclosed outside the standard intrapelvic lymph node templates, emphasizing the importance of pre-therapeutic imaging for both surgery and radiation therapy planning. Our results are similar to the proPSMA study in which changes in patient management were reported in 17.7% of high-risk patients [19].

Our study has a number of limitations. While sensitivity was high on a per-patient basis, mapping of intra-prostatic cancer to histologic specimens could not be reported on an octant basis as intended due to challenges of co-registering imaging and surgical specimens. Additionally, routine PSMA-immunohistochemistry was not part of the study but could provide further information on the extent to which lesional PSMA-expression contributes to the lack of detection. For similar reasons, mismatches between (mainly adjacent) histologically positive lymph nodes and their location on PET/CT are difficult to avoid. In addition, PSMA-positive nodes in other than the eight pelvic templates have been noted by the independent blinded readers but have not been part of the primary endpoint for the template-based analysis. A standard of reference for assessing bone lesions was not implemented, and that combined with study-defined exclusions of patients with bone disease, limited the interpretation of bone findings in the study. Finally, this manuscript does not report results on the analysis of molecular and clinical biomarkers (exploratory secondary endpoint) but will be the subject of future investigation.

## Conclusion

^68^Ga-PSMA-11 is a safe radiopharmaceutical and, in the setting of primary staging of high-risk prostate cancer patients, leads to increased diagnostic accuracy with a high impact on clinical management. Furthermore, pre-operative ^68^Ga-PSMA-11 PET/CT will inform accurate patient counselling by identifying PSMA-positive disease outside the planned surgical or radiotherapy field which could result in better clinical outcomes.

## Supplementary Information

Below is the link to the electronic supplementary material.

Supplementary file1 (DOCX 26 KB)

Supplementary file2 (DOCX 20.5 KB)

Supplementary file3 (XLSX 10.3 KB)

## Data Availability

For research data can be retrieved from the DFKZ, Heidelberg, Germany.

## Declarations

### Study registrations

EudraCT-Nr. 2016–001815-19,

ClinicalTrials.gov ID NCT03362359.

DRKS ID DRKS00013701.

## References

[1] Sung H, Ferlay J, Siegel RL, Laversanne M, Soerjomataram I, Jemal A, et al. Global cancer statistics 2020: GLOBOCAN estimates of incidence and mortality worldwide for 36 cancers in 185 countries. CA Cancer J Clin. 2021;71:209–49. 10.3322/caac.21660. 33538338 10.3322/caac.21660

[2] National Comprehensive Cancer Network (NCCN). Prostate cancer (Version 3.2024). 2024. https://pubmed.ncbi.nlm.nih.gov/38626801/

[3] Hövels AM, Heesakkers RAM, Adang EM, Jager GJ, Strum S, Hoogeveen YL, et al. The diagnostic accuracy of CT and MRI in the staging of pelvic lymph nodes in patients with prostate cancer: a meta-analysis. Clin Radiol. 2008;63:387–95. 10.1016/j.crad.2007.05.022. 18325358 10.1016/j.crad.2007.05.022

[4] Briganti A, Abdollah F, Nini A, Suardi N, Gallina A, Capitanio U, et al. Performance characteristics of computed tomography in detecting lymph node metastases in contemporary patients with prostate cancer treated with extended pelvic lymph node dissection. Eur Urol. 2012;61:1132–8. 10.1016/j.eururo.2011.11.008. 22099610 10.1016/j.eururo.2011.11.008

[5] Afshar-Oromieh A, Hetzheim H, Kübler W, Kratochwil C, Giesel FL, Hope TA, et al. Radiation dosimetry of (68)Ga-PSMA-11 (HBED-CC) and preliminary evaluation of optimal imaging timing. Eur J Nucl Med Mol Imaging. 2016;43:1611–20. 10.1007/s00259-016-3419-0. 27260521 10.1007/s00259-016-3419-0

[6] Afshar-Oromieh A, Malcher A, Eder M, Eisenhut M, Linhart HG, Hadaschik BA, et al. PET imaging with a [68Ga]gallium-labelled PSMA ligand for the diagnosis of prostate cancer: biodistribution in humans and first evaluation of tumour lesions. Eur J Nucl Med Mol Imaging. 2013;40:486–95. 10.1007/s00259-012-2298-2. 23179945 10.1007/s00259-012-2298-2

[7] Eder M, Schäfer M, Bauder-Wüst U, Hull W-E, Wängler C, Mier W, et al. 68Ga-complex lipophilicity and the targeting property of a urea-based PSMA inhibitor for PET imaging. Bioconjug Chem. 2012;23:688–97. 10.1021/bc200279b. 22369515 10.1021/bc200279b

[8] Afshar-Oromieh A, Avtzi E, Giesel FL, Holland-Letz T, Linhart HG, Eder M, et al. The diagnostic value of PET/CT imaging with the (68)Ga-labelled PSMA ligand HBED-CC in the diagnosis of recurrent prostate cancer. Eur J Nucl Med Mol Imaging. 2015;42:197–209. 10.1007/s00259-014-2949-6. 25411132 10.1007/s00259-014-2949-6 PMC4315487

[9] Afshar-Oromieh A, Zechmann CM, Malcher A, Eder M, Eisenhut M, Linhart HG, et al. Comparison of PET imaging with a ^68^Ga-labelled PSMA ligand and ^18^F-choline-based PET/CT for the diagnosis of recurrent prostate cancer. Eur J Nucl Med Mol Imaging. 2014;41:11–20. 10.1007/s00259-013-2525-5. 24072344 10.1007/s00259-013-2525-5 PMC3843747

[10] Fendler WP, Calais J, Eiber M, Flavell RR, Mishoe A, Feng FY, et al. Assessment of ^68^Ga-PSMA-11 PET accuracy in localizing recurrent prostate cancer: a prospective single-arm clinical trial. JAMA Oncol. 2019;5:856–63. 10.1001/jamaoncol.2019.0096. 30920593 10.1001/jamaoncol.2019.0096 PMC6567829

[11] FDA. FDA Approves First PSMA-Targeted PET Imaging Drug for Men with Prostate Cancer. 2020. https://www.fda.gov/drugs/news-events-human-drugs/fda-approves-second-psma-targeted-pet-imaging-drug-men-prostate-cancer

[12] EAU Guidelines on Prostate Cancer. Edn. presented at the EAU Annual Congress Milan. 2023. https://pubmed.ncbi.nlm.nih.gov/38614820/

[13] Fendler WP, Eiber M, Beheshti M, Bomanji J, Calais J, Ceci F, et al. PSMA PET/CT: joint EANM procedure guideline/SNMMI procedure standard for prostate cancer imaging 2.0. Eur J Nucl Med Mol Imaging. 2023;50:1466–86. 10.1007/s00259-022-06089-w. 36604326 10.1007/s00259-022-06089-w PMC10027805

[14] Surasi DS, Eiber M, Maurer T, Preston MA, Helfand BT, Josephson D, et al. Diagnostic performance and safety of positron emission tomography with 18F-rhPSMA-7.3 in patients with newly diagnosed unfavourable intermediate- to very-high-risk prostate cancer: results from a phase 3, prospective, multicentre study (LIGHTHOUSE). Eur Urol. 2023;84:361–70. 10.1016/j.eururo.2023.06.018. 37414702 10.1016/j.eururo.2023.06.018

[15] Giesel FL, Hadaschik B, Cardinale J, Radtke J, Vinsensia M, Lehnert W, et al. F-18 labelled PSMA-1007: biodistribution, radiation dosimetry and histopathological validation of tumor lesions in prostate cancer patients. Eur J Nucl Med Mol Imaging. 2017;44:678–88. 10.1007/s00259-016-3573-4. 27889802 10.1007/s00259-016-3573-4 PMC5323462

[16] Giesel FL, Knorr K, Spohn F, Will L, Maurer T, Flechsig P, et al. Detection efficacy of (18)F-PSMA-1007 PET/CT in 251 patients with biochemical recurrence of prostate cancer after radical prostatectomy. J Nucl Med. 2019;60:362–8. 10.2967/jnumed.118.212233. 30042163 10.2967/jnumed.118.212233 PMC6424235

[17] Sterzing F, Kratochwil C, Fiedler H, Katayama S, Habl G, Kopka K, et al. (68)Ga-PSMA-11 PET/CT: a new technique with high potential for the radiotherapeutic management of prostate cancer patients. Eur J Nucl Med Mol Imaging. 2016;43:34–41. 10.1007/s00259-015-3188-1. 26404016 10.1007/s00259-015-3188-1 PMC4771815

[18] Calais J, Ceci F, Eiber M, Hope TA, Hofman MS, Rischpler C, et al. (18)F-fluciclovine PET-CT and (68)Ga-PSMA-11 PET-CT in patients with early biochemical recurrence after prostatectomy: a prospective, single-centre, single-arm, comparative imaging trial. Lancet Oncol. 2019;20:1286–94. 10.1016/s1470-2045(19)30415-2. 31375469 10.1016/S1470-2045(19)30415-2 PMC7469487

[19] Hofman MS, Lawrentschuk N, Francis RJ, Tang C, Vela I, Thomas P, et al. Prostate-specific membrane antigen PET-CT in patients with high-risk prostate cancer before curative-intent surgery or radiotherapy (proPSMA): a prospective, randomised, multicentre study. Lancet. 2020;395:1208–16. 10.1016/s0140-6736(20)30314-7. 32209449 10.1016/S0140-6736(20)30314-7

[20] Eiber M, Herrmann K, Calais J, Hadaschik B, Giesel FL, Hartenbach M, et al. Prostate cancer molecular imaging standardized evaluation (PROMISE): proposed mitnm classification for the interpretation of PSMA-ligand PET/CT. J Nucl Med. 2018;59:469–78. 10.2967/jnumed.117.198119. 29123012 10.2967/jnumed.117.198119

[21] Koerber SA, Utzinger MT, Kratochwil C, Kesch C, Haefner MF, Katayama S, et al. (68)Ga-PSMA-11 PET/CT in newly diagnosed carcinoma of the prostate: correlation of intraprostatic PSMA uptake with several clinical parameters. J Nucl Med. 2017;58:1943–8. 10.2967/jnumed.117.190314. 28619734 10.2967/jnumed.117.190314

[22] Maurer T, Gschwend JE, Rauscher I, Souvatzoglou M, Haller B, Weirich G, et al. Diagnostic efficacy of (68)gallium-PSMA positron emission tomography compared to conventional imaging for lymph node staging of 130 consecutive patients with intermediate to high risk prostate cancer. J Urol. 2016;195:1436–43. 10.1016/j.juro.2015.12.025. 26682756 10.1016/j.juro.2015.12.025

[23] Harisinghani MG, Barentsz J, Hahn PF, Deserno WM, Tabatabaei S, van de Kaa CH, et al. Noninvasive detection of clinically occult lymph-node metastases in prostate cancer. N Engl J Med. 2003;348:2491–9. 10.1056/NEJMoa022749. 12815134 10.1056/NEJMoa022749

[24] Hope TA, Eiber M, Armstrong WR, Juarez R, Murthy V, Lawhn-Heath C, et al. Diagnostic accuracy of ^68^Ga-PSMA-11 PET for pelvic nodal metastasis detection prior to radical prostatectomy and pelvic lymph node dissection: a multicenter prospective phase 3 imaging trial. JAMA Oncol. 2021;7:1635–42. 10.1001/jamaoncol.2021.3771. 34529005 10.1001/jamaoncol.2021.3771 PMC8446902

[25] Pienta KJ, Gorin MA, Rowe SP, Carroll PR, Pouliot F, Probst S, et al. A phase 2/3 prospective multicenter study of the diagnostic accuracy of prostate specific membrane antigen PET/CT with (18)F-DCFPyL in prostate cancer patients (OSPREY). J Urol. 2021;206:52–61. 10.1097/ju.0000000000001698. 33634707 10.1097/JU.0000000000001698 PMC8556578

[26] Petersen LJ, Zacho HD. PSMA PET for primary lymph node staging of intermediate and high-risk prostate cancer: an expedited systematic review. Cancer Imaging. 2020;20:10. 10.1186/s40644-020-0290-9. 31973751 10.1186/s40644-020-0290-9 PMC6979382

[27] Rutjes AW, Reitsma JB, Di Nisio M, Smidt N, van Rijn JC, Bossuyt PM. Evidence of bias and variation in diagnostic accuracy studies. CMAJ. 2006;174:469–76. 10.1503/cmaj.050090. 16477057 10.1503/cmaj.050090 PMC1373751

[28] Jager GJ, Barentsz JO, Oosterhof GO, Witjes JA, Ruijs SJ. Pelvic adenopathy in prostatic and urinary bladder carcinoma: MR imaging with a three-dimensional TI-weighted magnetization-prepared-rapid gradient-echo sequence. Am J Roentgenol. 1996;167:1503–7. 10.2214/ajr.167.6.8956585. 8956585 10.2214/ajr.167.6.8956585

[29] Eiber M, Weirich G, Holzapfel K, Souvatzoglou M, Haller B, Rauscher I, et al. Simultaneous (68)Ga-PSMA HBED-CC PET/MRI improves the localization of primary prostate cancer. Eur Urol. 2016;70:829–36. 10.1016/j.eururo.2015.12.053. 26795686 10.1016/j.eururo.2015.12.053

[30] Wright GL Jr., Haley C, Beckett ML, Schellhammer PF. Expression of prostate-specific membrane antigen in normal, benign, and malignant prostate tissues. Urol Oncol. 1995;1:18–28. 10.1016/1078-1439(95)00002-y. 21224086 10.1016/1078-1439(95)00002-y

[31] Fendler WP, Schmidt DF, Wenter V, Thierfelder KM, Zach C, Stief C, et al. 68Ga-PSMA PET/CT detects the location and extent of primary prostate cancer. J Nucl Med. 2016;57:1720–5. 10.2967/jnumed.116.172627. 27261520 10.2967/jnumed.116.172627

[32] Pizzuto DA, Müller J, Mühlematter U, Rupp NJ, Töpfer A, Mortezavi A, et al. The central zone has increased 68Ga-PSMA-11 uptake: “Mickey Mouse ears” can be hot on 68Ga-PSMA-11 PET. Eur J Nucl Med Mol Imaging. 2018;45:1335–43. 10.1007/s00259-018-3979-2. 29523924 10.1007/s00259-018-3979-2

[33] Fendler WP, Calais J, Allen-Auerbach M, Bluemel C, Eberhardt N, Emmett L, et al. (68)Ga-PSMA-11 PET/CT interobserver agreement for prostate cancer assessments: an international multicenter prospective study. J Nucl Med. 2017;58:1617–23. 10.2967/jnumed.117.190827. 28408531 10.2967/jnumed.117.190827

[34] Pyka T, Okamoto S, Dahlbender M, Tauber R, Retz M, Heck M, et al. Comparison of bone scintigraphy and (68)Ga-PSMA PET for skeletal staging in prostate cancer. Eur J Nucl Med Mol Imaging. 2016;43:2114–21. 10.1007/s00259-016-3435-0. 27290607 10.1007/s00259-016-3435-0

[35] Langsteger W, Haim S, Knauer M, Waldenberger P, Emmanuel K, Loidl W, et al. Imaging of bone metastases in prostate cancer: an update. Q J Nucl Med Mol Imaging. 2012;56:447–58. 23069924

