# Supplementary material for: The German Cancer Consortium (DKTK) multi-center prospective phase 1/2 68Ga-PSMA-11 PET-imaging trial in newly-diagnosed high-risk prostate cancer: Safety and diagnostic accuracy compared to histopathology and their impact on patient management
Source: Eur J Nucl Med Mol Imaging. 2025 Nov 15;53(5):2994–3006. doi: 10.1007/s00259-025-07540-4 (PMC13013323; doi:10.1007/s00259-025-07540-4)
Supplement: Supplementary file 1 — Supplementary file1 (DOCX 26 KB) [file 259_2025_7540_MOESM1_ESM.docx]

**Supplementary Tables**

Supplementary table 1. Distribution of patients by site.

| **Site**  **number** | **Site name**  **and country** | **Patient screened / enrolled (n)** | | **Patients excluded (n)** | | **Patients**  **treated (n)** | |  |
| --- | --- | --- | --- | --- | --- | --- | --- | --- |
| 01 | Dresden, Germany | 11 | | 0 | | 11 | |  |
| 03 | Essen, Germany | 2 | | 0 | | 2 | |  |
| 04 | Heidelberg, Germany | 53 | | 2 | | 51 | |  |
| 05 | Tübingen, Germany | 9 | | 0 | | 9 | |  |
| 06 | Freiburg, Germany | 19 | | 4 | | 15 | |  |
| 08 | TU Munich, Germany | 84 | | 3 | | 81 | |  |
| 09 | Erlangen, Germany | 0 | | 0 | | 0 | |  |
| 10 | Innsbruck, Austria | 3 | | 0 | | 3 | |  |
| 11 | Zurich, Switzerland | 1 | | 0 | | 1 | |  |
| **Total** | | | **182** | | **9** | | **173** | |

**Supplementary Table 2. Sensitivity of 68Ga-PSMA11 PET in relation to size of histopathological lymph node infiltation (LNI)**

| Histopathological size | N | Sens |  | 95% CI |
| --- | --- | --- | --- | --- |
| No threshold / all patients | 55 | 0.400 |  | 0.271 – 0.529 |
|  |  |  |  |  |
| Largest extent of disease (in mm) | | | | |
| >2 | 48 | 0.458 |  | 0.314 – 0.608 |
| **>3** | **39** | **0.564** |  | **0.396 – 0.722** |
| **>4** | **37** | **0.595** |  | **0.421 – 0.752** |
| **>5** | **29** | **0.690** |  | **0.492 – 0.847** |
| >6 | 25 | 0.680 |  | 0.465 – 0.851 |
| >7 | 17 | 0.824 |  | 0.566 – 0.962 |
| >8 | 14 | 0.857 |  | 0.572 – 0.982 |
|  |  |  |  |  |
| Smallest extent of disease (in mm) | | | | |
| >2 | 35 | 0.571 |  | 0.394 – 0.737 |
| **>3** | **27** | **0.630** |  | **0.424 – 0.806** |
| **>4** | **20** | **0.750** |  | **0.509 – 0.913** |
| **>5** | **17** | **0.824** |  | **0.566 – 0.962** |
| >6 | 15 | 0.800 |  | 0.519 – 0.957 |
| >7 | 10 | 0.800 |  | 0.444 – 0.975 |
| >8 | 9 | 0.778 |  | 0.400 – 0.972 |
|  |  |  |  |  |
|  |  |  |  |  |

Data in bold indicates treshold of disease size above resolution in PET („diagnostic window in PET“).

Supplementary Table 3: Treatment emergent adverse events by severity grade (Safety Set)

| **System Organ Class** | **Preferred Term** | **Total (N=173)**  **(n [%])** |
| --- | --- | --- |
| Toxicity Grade 2 | | |
| Cardiac disorders | Total | 1 (0.6%) |
|  | Sinus arrhythmia | 1 |
| Toxicity Grade 1 | | |
| Investigations | Total | 10 (5.8%) |
|  | Alanine aminotransferase increased | 1 |
|  | Aspartate aminotransferase increased | 1 |
|  | Blood creatine increased | 1 |
|  | Blood glucose increased | 1 |
|  | Blood urea increased | 1 |
|  | Gamma-glutamyl transferase increased | 1 |
|  | Liver function test increased | 1 |
|  | Monocyte count increased | 1 |
|  | Neutrophil count increased | 1 |
|  | White blood cell count increased | 1 |
| Cardiac disorders | Total | 4 (2.3%) |
|  | Atrial fibrillation | 3 |
|  | Tachycardia | 1 |
| Musculoskeletal and connective tissue disorders | Total | 2 (1.2%) |
|  | Muscle spasms | 1 |
|  | Pain in extremity | 1 |
| Nervous system disorders | Total | 2 (1.2%) |
|  | Headache | 1 |
|  | Hypertonia | 1 |
| Skin and subcutaneous tissue disorders | Total | 1 (0.6%) |
|  | Erythema | 1 |

Only the most severe toxicity grade occurrence within each SOC and PT for each patient was counted

Supplementary Table 4. Bone lesion assessment for positive patients on bone scintigraphy or 68a-PSMA11 PET/CT

|  | | | **68Ga-PSMA11** | | | **Bone scintigraphy** | |
| --- | --- | --- | --- | --- | --- | --- | --- |
| **Patient** | **Location** | **Final state** | | **No. bone Lesions** | **Final state** | | **No. bone lesions** |
| 04-015 | Lumbar spine | Negative | | 0 | Positive | | 1 |
| 04-030 | Pelvis | Negative | | 0 | Positive | | 1 |
| 04-037 | Thoracic spine | Negative | | 0 | Positive | | 1 |
| 05-007 | Pelvis | Negative | | 0 | Positive | | 1 |
| 06-003 | Clavicula/scapula | Negative | | 0 | Positive | | 1 |
| 08-031 | Pelvis | Negative | | 0 | Positive | | 1 |
| 08-056 | Ribs/sternum | Negative | | 0 | Positive | | 1 |
| 04-041* | Pelvis | Positive | | 1 | Positive | | 1 |
| 08-049 | Lumbar spine | Positive | | 1 | Negative | | 0 |
| 08-049 | Pelvis | Positive | | 1 | Negative | | 0 |
| 08-049 | Ribs/sternum | Positive | | 1 | Negative | | 0 |
| 08-049 | Skull | Positive | | 1 | Negative | | 0 |
| 08-049 | Thoracic spine | Positive | | 1 | Equivocal^§^ | | 1 |
| 08-065 | Thoracic spine | Positive | | 1 | Negative | | 0 |
| 08-074 | Ribs/sternum | Positive | | 1 | Negative | | 0 |
| 08-074 | Thoracic spine | Positive | | 1 | Negative | | 0 |
| 08-077 | Pelvis | Positive | | 1 | Equivocal^§^ | | 1 |

* patient with concordant positive finding in same region

§ equivocal regions were counted as negative
