# Supplementary material for: The German Cancer Consortium (DKTK) multi-center prospective phase 1/2 68Ga-PSMA-11 PET-imaging trial in newly-diagnosed high-risk prostate cancer: Safety and diagnostic accuracy compared to histopathology and their impact on patient management
Source: Eur J Nucl Med Mol Imaging. 2025 Nov 15;53(5):2994–3006. doi: 10.1007/s00259-025-07540-4 (PMC13013323; doi:10.1007/s00259-025-07540-4)
Supplement: Supplementary file 2 — Supplementary file2 (DOCX 20.5 KB) [file 259_2025_7540_MOESM2_ESM.docx]

**Appendix / Supplementary Material**

Appendix 1.

Study protocol

Appendix 2.

Subject imaging manual

**Supplementary Material**

**Material and Methods / Procedures**

The mass dose of Ga-68-PSMA-11 was ≤ 6 µg (micro-dose), labelled with 150 (± 50) MBq ^68^Ga per dose. PSMA-11 is labelled with gallium-68 according to Good Radiopharmaceutical Practice (GRPP) per European Association of Nuclear Medicine (EANM) guidelines and according to national regulations on radiopharmaceuticals. Each radio pharmacy underwent training and specific written instructions on the labelling procedure, in concordance with the Investigational Medicinal Product Dossier (IMPD), to ensure a comparable labelling efficiency (radiochemical yield) across all sites. ^1,2^

**Material and Methods / Surgery, follow-up and Histopathology Correlation**

Predefined subregions:

1. external iliac artery and vein, 2. obturator fossa (located (cranially and caudally to the obturator nerve), 3. medial and lateral to the internal iliac artery, 4. common iliac lymph nodes up ureteric crossing (each left and right).

**Material and Methods / Statistical analysis:**

True positives were defined as regions that were concordantly positive by two readers, based on majority rule, and/or were positive based on histopathology. Regions demonstrating positive imaging, but negative pathology findings were considered as false positives. Regions, which were negative on imaging but had positive pathology were considered false negatives and lastly regions negative on imaging and pathology were assessed as true negatives.

The results are presented as Cohen’s Kappa as well as Brennan/Prediger Kappa. The Brennan/Prediger Kappa was introduced since for prostate assessment at the patient level, one reader did not have negative regions due to the study design. Therefore, the Cohen’s Kappa resulted in 𝜿=0. The Brendon/Prediger Kappa corrects for prevalence and bias. Except for the patient-level prostate assessment, the Kappa values reported within the text always refers to Cohen’s Kappa.

**Interreader agreement – adjudication**

For prostate assessment on the quadrant level, adjudication was required in 87 of 140 patients. In nine of 140 cases, adjudication for N-staging was required. In four cases, the discrepancy was between one versus multiple lymph nodes, while the other five cases were disagreements between no positive lymph nodes and at least one positive lymph node . On a subregion-level, adjudication was triggered in 30 patients with discrepant evaluations in 65 subregions from a total of 1260 subregions. In three of 140 cases, adjudication for M-staging was required. On a subregion-level, adjudication was required in 15 patients in 20 subregions out of 1260. Only in three cases was a discrepancy between positive versus negative observations recorded. The other cases were discrepancies between positive versus equivocal or negative versus equivocal observations.

**References**

1. Afshar-Oromieh A, Zechmann CM, Malcher A, et al. Comparison of PET imaging with a 68Ga-labelled PSMA ligand and 18F-choline-based PET/CT for the diagnosis of recurrent prostate cancer. *European Journal of Nuclear Medicine and Molecular Imaging* 2014; **41**(1): 11-20.

2. Afshar-Oromieh A, Haberkorn U, Schlemmer HP, et al. Comparison of PET/CT and PET/MRI hybrid systems using a 68Ga-labelled PSMA ligand for the diagnosis of recurrent prostate cancer: initial experience. *Eur J Nucl Med Mol Imaging* 2014; **41**(5): 887-97.
